# Sicien H. Chen, one of the pioneers and founders of Chinese entomology

**DOI:** 10.1007/s13238-021-00829-8

**Published:** 2021-03-04

**Authors:** Yejing Ge

**Affiliations:** Department for the History of Science and Scientific Archaeology, University of Science and Technology of China, Hefei, 230026 China

**Figure 1:**
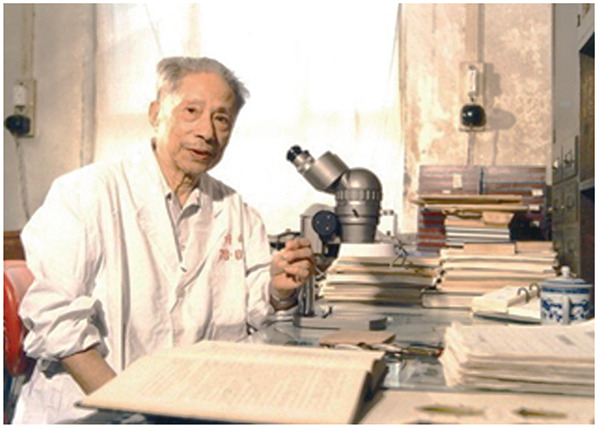
Sicien H. Chen (1905–1988)

Sicien H. Chen (陈世骧, 1905–1988) was an academician of the Chinese Academy of Sciences (CAS) and a distinguished biologist, entomologist, and evolutionary taxonomist (Fig. 1). As a meticulous and diligent scientist, he discovered 76 new genera, 949 new species, and contributed more than 180 publications, including articles, monographs, and conference proceedings. Sicien H. Chen has been included in the *Who*’*s Who* of various countries and continues to be highly regarded by the international scientific community.

**Figure 2:**
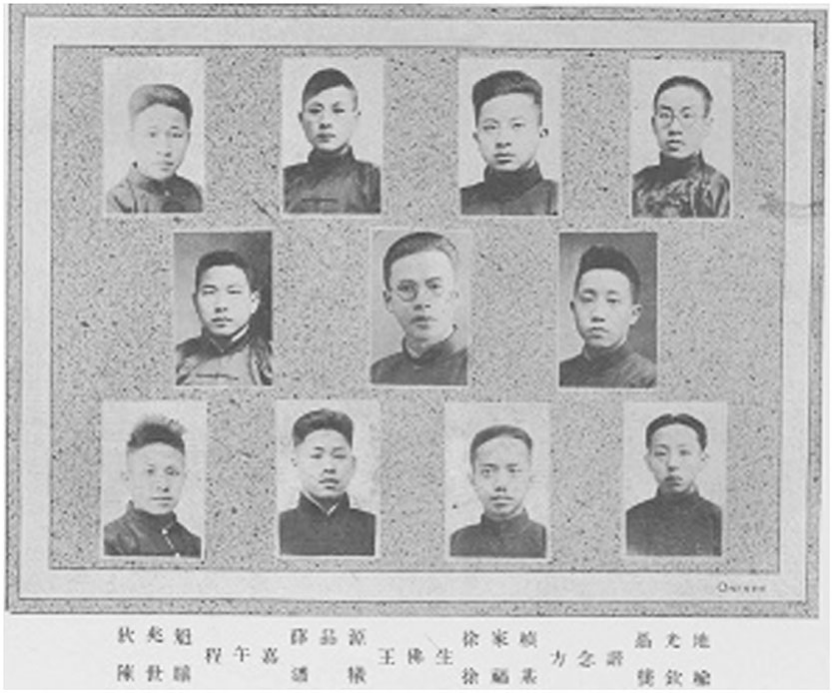
Sicien H. Chen (on the left bottom) and his classmates at Fudan University in 1924

Sicien H. Chen was born into a scholarly family in Jiaxing, Zhejiang Province: a fertile area rampant with rice borer infestation. To scientifically guard against the major threat of pests, his father, Chih-Kung Chen (陈志巩), initiated the first folk pest control organization, named the Committee of Borer Control. When the May 4th Movement broke out, Chen, a student at the Second Higher Primary School, performed a metaphorical play with his teachers in the street (Zhang, 1988). Thereafter, the seed of saving the nation with science was planted. Supported by this belief, he enrolled at the Biology Department of Fudan University in 1924, where he demonstrated great diligence and eagerness (Fig. 2). Coincidentally, Auguste Savio, an entomologist and member of the French Entomological Society, taught here during his undergraduate period. After graduation in 1928, Chen continued with his academic career in entomology and became a doctoral candidate at the National Museum of Natural History in Paris as a result of his excellent performance. Notably, Sicien H. Chen and Yon-Yon Zia (谢蕴贞) were engaged in France on January 15, 1933. Subsequently, he finished his dissertation on *Recherches sur les Chrysomelinae de la Chine et du Tonkin*, which became a classic in the history of entomology and was awarded the Prix Passet in 1935, and he received his doctoral degree a year later (Chen, 1934). There were numerous opportunities to work in chief institutions such as the British Museum, National Museum of Natural History, and German Entomological Institute, but as an ardent patriot, he eventually returned to China and dedicated himself to entomology. He successively served as a researcher at the Institute of Botany and Zoology in August 1934 and the Institute of Zoology in May 1944 at the Academia Sinica. After the founding of the People’s Republic of China, he was designated as the director of the Laboratory of Entomology (1950–1953), the Institute of Entomology (1953–1962), and the Institute of Zoology (1962–1982). In 1955, he was elected as a member of the First Committee Member of Academic Divisions, now jointly known as the academician.

**Figure 3:**
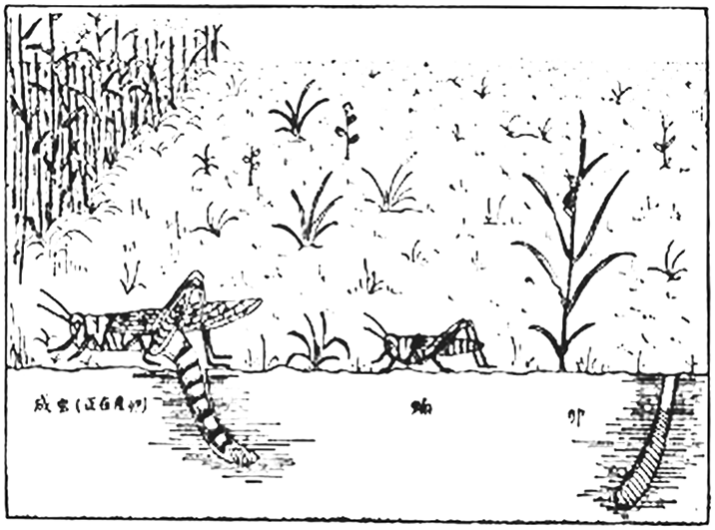
An illustration of East Asian migratory locusts in the 1950s

During the spring of 1952, many bacteria-carrying insects were discovered in Northeast China, which was inimical to the whole country both socially and economically. After learning of the outbreak, Sicien H. Chen undertook entomological research and identified a variety of specimens of these insects in a scientific investigation of anti-bacteriological warfare. Almost simultaneously, locust plagues broke out with great intensity (Fig. 3). In early 1952, he had taken the initiative to establish a locust research group in Shanghai, where he bred East Asian migratory locusts to study their fertility. Meanwhile, in the following year, after noticing the significance of team construction, he formally divided the Institute of Entomology into 12 research directions. Many highly specialized scientists were active, including Hung-Fu Chu (朱弘复), Yu-Su Liu (刘玉素), Shih-Chun Ma (马世骏), Chun-Teh Chin (钦俊德), Chin-Jen Luh (陆近仁), Chung-Lo Liu (刘崇乐), Pang-Hua Tsai (蔡邦华), Lan-Chou Feng (冯兰洲), Yao Hsiung (熊尧), Kwen-Yuan Kung (龚坤元), Yung-Chang Chao (赵养昌), and so on (Institute of Zoology, 2008). The members, as well as other state-owned organizations, were engaged in basic studies of locusts. As a corollary, he successfully controlled the ravages of the locusts and solved the millennium locust problem by collaborating with his team, based on the thoughtful and profound idea of combining basic research, interdisciplinary collaboration, and existing methods. A series of innovative breakthroughs by these elites were affirmed by Premier Zhou Enlai, who decided to construct a building for the newly established institute (Li, 2020), and their penetrating study on *Ecology and Physiology of East Asian Migratory Locust and Their Significance in the Eradication of Locust Pests* won the second prize at the State Natural Science Award in 1982.

**Figure 4:**
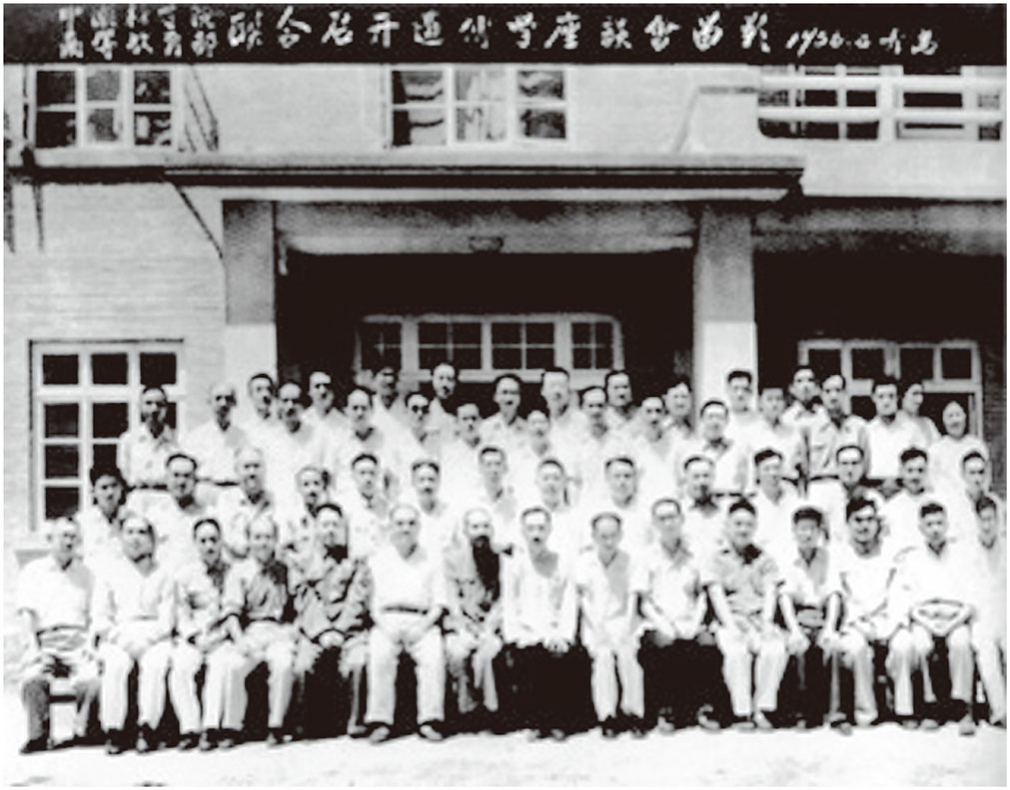
The Qingdao Meeting on Genetics in August 1956

On the path of scrupulous scholarship, Sicien H. Chen had the courage to seek the truth from the facts and persevere. In August 1956, the Ministry of Higher Education and CAS jointly convened the Qingdao Meeting on Genetics (Fig. 4) to implement the policy of “letting a hundred flowers blossom and a hundred schools of thought contend” (双百方针). At the meeting, Chen offered four criticisms of Lysenko’s new insights on species formation, which corrected the ideological bias, set a model for academic research, and laid a solid foundation for further research in the realm of genetics. Moreover, he insisted on a self-understanding of “zhengming” (争鸣); that is, it must be based on theory and facts and “said with solid judgment; “opposed to labeling at random, criticizing “concepts” with “concepts” and all monastic rules (Chen, 1957).

**Figure 5:**
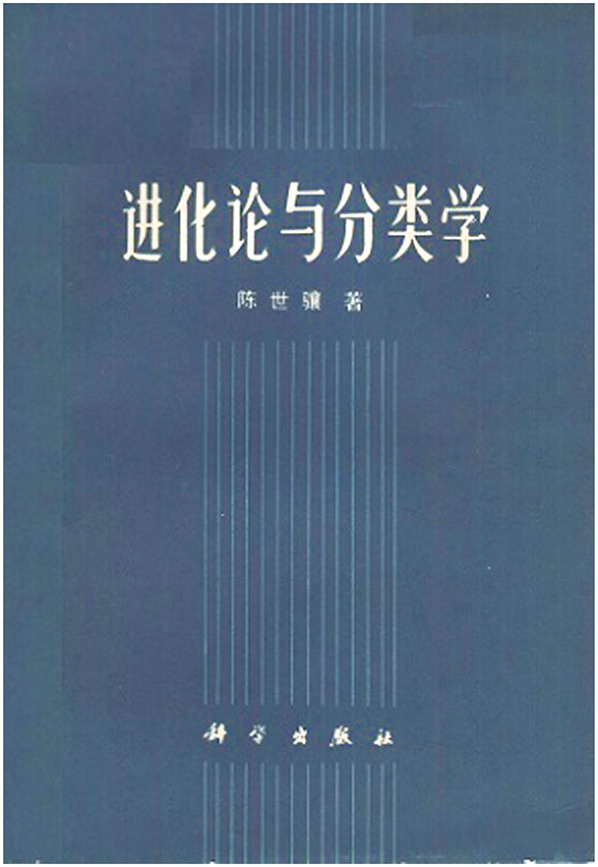
*Evolution and Taxonomy*, by Sicien H. Chen in 1978

**Figure 6:**
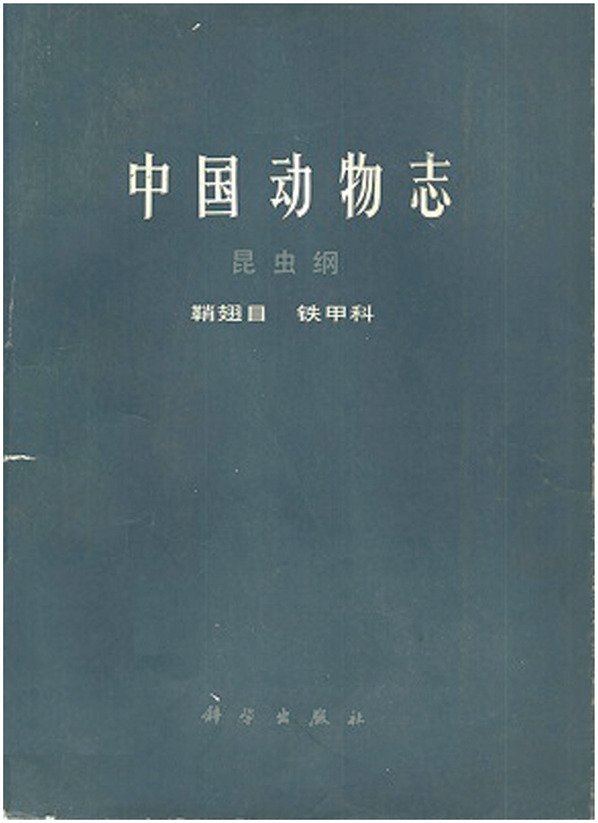
*Fauna Sinica Insecta Coleoptera Hispidae*, by Sicien H. Chen et al. in 1986

As a zoological discipline, entomology typically consists of taxonomic evolutionary, behavioral, morphological, physiological, and genetic research in scientific practice. In a similar vein, Sicien H. Chen had always been an enthusiastic and indefatigable scientist, working on the frontiers of Chrysomelidae (Coleoptera), taxonomy, biological evolution, and so on. Particularly, the taxonomic system was conceptualized as a microcosm of evolutionary history. In the book *Evolution and Taxonomy* (Fig. 5), Sicien H. Chen systematically described three fundamental issues, including the concept of species, phylogeny, and characterization, to create a theoretical system of evolutionary taxonomy that pushed biological taxonomy to a new level. Moreover, he concentrated on this meshing of natural philosophy with biology, proposed “change and unchanged, adaptive and non-adaptive” species, expounded “ten major events in the history of biological evolution”, and elaborated on the three paths of biological evolution, which prominently contributed to the enrichment and development of Darwinism (Chen, 1978). He creatively divided biological evolution into Superkingdom Acytonia, Superkingdom Procaryota (Kingdom Mycomonera and Kingdom Phycomonera), and Superkingdom Eucaryota (Kingdom Plantae, Kingdom Fungi, and Kingdom Animalia) (Chen SH and Chen SY, 1979). He was the editor-in-chief of the book *Fauna Sinica Insecta Coleoptera Hispidae* (Fig. 6), which won first prize at the Scientific and Technological Progress Award of CAS in 1988 and second prize at the State Natural Science Award in 1989, boosting biology and zoology education in China (Chen et al., 1986).

He was among the pioneer advocates of entomology institutionalization in China. In June 1937, a group of ambitious entomological scholars, led by Sicien H. Chen, were poised to establish the Chinese Entomological Society with the support of Chenfu F. Wu (胡经甫) and We-I Yang (杨惟义) (Science, 1937). Unfortunately, this project was temporarily interrupted due to the outbreak of the Anti-Japanese War. In May 1944, more than 30 biologists, including Sicien H. Chen, actively applied for the preparation of the society once again, and it was ultimately established on October 12. In the following year, Sicien H. Chen was the vice-chairman of the Chinese Agricultural Society in 1960 and chairman of the Chinese Entomological Society in 1978. Accordingly, he founded Zoological Systematics in 1964 and served as an editorial board member of journals such as *Acta Entomologica Sinica*, *Entomotaxonomia*, and *the Chinese Science Bulletin* (Lu et al., 1994). As such, these societies and journals still act as productive resources, and have had profound international influence so far. Sicien H. Chen joined the Jiu San Society (九三学社) in 1952 and the Communist Party of China in 1979. In March 1980, 38 members, represented by Sicien H. Chen, strongly urged for the restoration and reinforcement of biology teaching at the Second Congress of the National Association for Science and Technology.

Over the decades, Sicien H. Chen was deeply aware that international cooperation and exchange were not only the historical tradition of Chinese science but also the irresistible trend of global science. Around the 1970s, the research-related factors were significantly altered due to the national and international circumstances fluctuating in this period, making extensive scientific exchanges possible. Thus, Sicien H. Chen was able to make his projects more international. In 1973, Prof. Saburo Tamura of the Department of Agriculture, University of Tokyo visited the Institute of Zoology. In May 1975, Prof. De Wilde, a renowned entomologist in the Netherlands, was invited for academic visits. In July of the same year, he effectively communicated with a 10-member US delegation on integrated pest control, accompanied by his colleagues. In September 1979, Sicien H. Chen and Shih-Chun Ma met with Dr. Ching-Hsi Chao (赵景熹), a Chinese-American entomologist (Institute of Zoology, 2008). In 1980, Sicien H. Chen received a 32-member delegation of the American Entomological Society. During this period, they gave several academic lectures on pest management, forest pest control, and feeding of predatory insects. In 1984, Sicien H. Chen, then nearly 80 years old, delivered the lecture, *Evolution and Classification of the Chrysomelid Beetles*, at the 17th International Congress of Entomology that was held in Hamburg, Germany with 2,500 participants, which was appreciated by scholars at the conference (Lv, 2008).

On January 25, 1988, Sicien H. Chen passed away. He kept discussing *Fauna Sinica* with his colleagues until the day before his hospitalization. Although he has left us, his patriotic duty, strategic wisdom, rigorous approach, cooperative spirit, and opening consciousness have not disappeared and are worthy of appreciation and learning by all scientists. In his later years, he succinctly wrote (Li, 1989):

An old scientist advances cautiously (科研老卒, 意在过河).

A lifelong career will always be (生命不已, 壮志难磨).

Trifling as the trickle is, it makes a mickle (涓流虽小, 积少成多).

The sunset shining in the sky illuminates the clear waves (晚霞万里, 照澈清波).

It is likewise the summary of the practice of airing aspirations for all his life.
